# Anatomical Predictors of Valve Malposition During Self-Expandable Transcatheter Aortic Valve Replacement

**DOI:** 10.3389/fcvm.2021.600356

**Published:** 2021-07-12

**Authors:** Jie Li, Yinghao Sun, Shengneng Zheng, Guang Li, Haojian Dong, Ming Fu, Yujing Mo, Yi Li, Huadong Liu, Zhaoyan Xu, Liting Zhang, Yong Cao, Ruixin Fan, D. Scott Lim, Jianfang Luo

**Affiliations:** ^1^Guangdong Cardiovascular Institute, Guangdong Provincial People's Hospital, Guangdong Academy of Medical Sciences, Guangzhou, China; ^2^The First Affiliated Hospital of Sun Yat-sen University, Guangzhou, China; ^3^Shenzhen People's Hospital, Shenzhen, China; ^4^The First People Hospital of Foshan, Foshan, China; ^5^Zhongshan City People's Hospital, Zhongshan, China; ^6^Gaozhou People's Hospital, Gaozhou, China; ^7^University of Virginia Health System Hospital, Virginia, NV, United States

**Keywords:** computed tomography, malposition, predictor, self-expandable, transcatheter aortic valve replacement

## Abstract

**Background:** The consequence of valve malposition (VM) during transcatheter aortic valve replacement (TAVR) can be severe, but the determinants of VM with self-expandable TAVR have not been thoroughly evaluated. We aimed to investigate the anatomical predictors of VM during self-expandable TAVR.

**Methods:** In this multicenter retrospective study, TAVR was performed using the Venus A-Valve. The baseline, computed tomography, and procedural characteristics along with clinical outcomes were collected. Multivariate logistic regression model and receiver operating characteristic (ROC) curve analyses were performed.

**Results:** A total of 84 consecutive patients (23 with VM) were included. Stepwise regression showed that annulus perimeter/left ventricular outflow tract perimeter (AL ratio) and sinotubular junction (STJ) height were predictors of VM. The ROC curve indicated a moderate strength of AL ratio [area under the curve (AUC) 0.71, cutoff 0.96] and a weak strength of STJ height (AUC 0.69, cutoff 23.8 mm) to predict VM. The combination of both predictors revealed a higher predictive value of VM (AUC 0.77). In multivariate analysis, AL ratio <0.96 [odds ratio (OR) 3.98, *p* = 0.015] and STJ height ≥23.8 mm (OR 4.63, *p* = 0.008) were strong independent predictors of VM. The presence of both predictors was associated with a very high risk of VM (OR 10.67, *p* = 0.002). The rate of moderate-to-severe paravalvular regurgitation was higher in patients with VM at 30 days (26.1 vs. 4.9%, *p* = 0.011).

**Conclusions:** A conical left ventricular outflow tract and tall aortic sinuses were strong anatomical predictors of VM during self-expandable TAVR.

## Introduction

Transcatheter aortic valve replacement (TAVR) has become an alternative to surgical aortic valve replacement in patients with symptomatic severe aortic stenosis (1–4). However, unlike standard open surgical aortic valve replacement in which the surgical valve is sewn into place, implantation of the transcatheter heart valve can incur the risk of malposition during TAVR, which if significant may lead to severe aortic regurgitation or valve embolization and potentially necessitate implantation of a second prosthesis. According to the Valve Academic Research Consortium-2 (VARC-2) definitions, valve malposition (VM) can be categorized as migration, embolization, or ectopic deployment (5). Despite a low incidence, VM has been related to a higher mortality after TAVR (6, 7). Several predictors of VM in patients undergoing TAVR have been reported, including highly calcified aortic valves and the use of self-expandable (as compared with balloon-expandable) prostheses (6, 7), but the anatomical features associated with VM in self-expandable TAVR have not been thoroughly evaluated and specifically not evaluated in the Chinese population, which tends to have a significantly higher calcium burden (8). Therefore, we aimed to further investigate the anatomical predictors of VM during self-expandable TAVR with the Venus A-Valve.

## Materials and Methods

### Study Design

In this multicenter retrospective study, TAVR was performed in patients with symptomatic severe aortic stenosis using the self-expandable Venus A-Valve (Venus MedTech, Hangzhou, China). The Venus A-Valve has a similar supra-annular design as the Medtronic CoreValve but stronger radial force at the inflow end, which may be advantageous in bicuspid anatomy and severe calcification (9). The access routes and size of the prostheses were determined by the individual heart teams based on multidetector computed tomography (MDCT). Standardized measurements on MDCT and on angiography were both performed by two independent investigators. Patients with optimal position and malposition of the prostheses were included, while those with suboptimal position of the prostheses were excluded. The baseline, MDCT, and procedural characteristics along with clinical outcomes were collected. This study was approved by the Research Ethics Committee of Guangdong Provincial People's Hospital (No. GDREC2019384H), with a waiver of informed consent.

### Definitions

**Figure 1:**
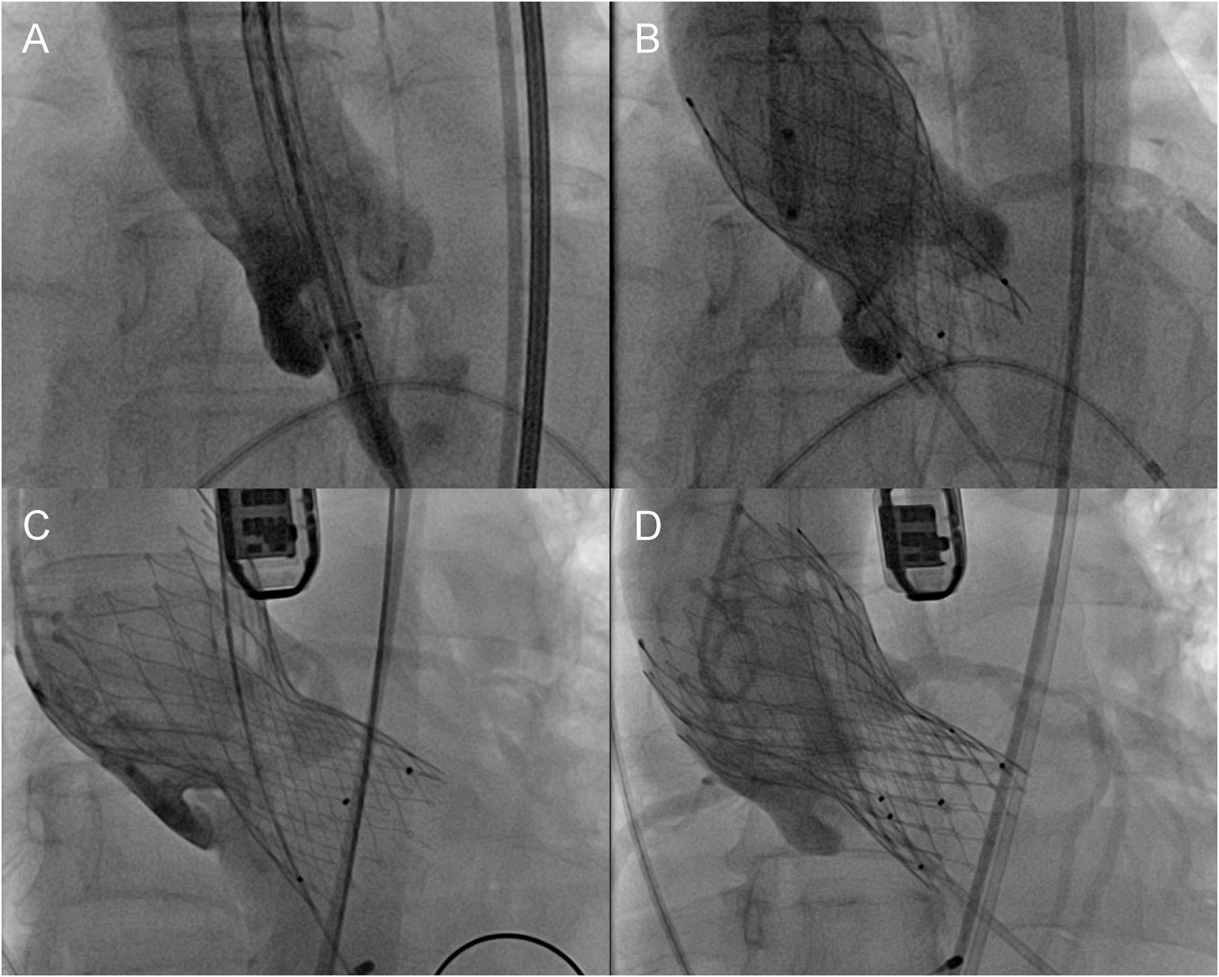
Definition of different positions of the prosthesis. **(A)** Initial position, **(B)** optimal position, **(C)** malposition, and **(D)** implantation of a second prosthesis.

The Venus A-Valve has three radiopaque markers at half a cell (5–6 mm) above the inflow end, indicating the optimal landing zone (Appendix 1). The default initial position of the markers was aligned with the annular plane. Optimal position on angiography was defined as the markers being within the range from 4 mm above to 8 mm below the annulus, such that the covered cells of the prosthesis remain in contact with the annulus. Suboptimal position denoted markers being outside the range of optimal position but with acceptable hemodynamics (less than moderate paravalvular regurgitation evaluated by angiography and transesophageal echocardiography). Malposition indicated severe migration of the first valve, resulting in unacceptable paravalvular regurgitation and implantation of a second valve. These definitions were modified according to VARC-2 criteria (Figure 1).

The primary outcome was all-cause mortality at 30 days. The secondary outcomes were VARC-2 defined complications at 30 days.

### Multidetector Computed Tomography

All MDCT images were analyzed by two independent investigators using 3 mensio software (Pie Medical, Bilthoven, The Netherlands). First, we needed to fine-tune the default central line of the aortic root. Second, the nadirs of all coronary sinuses, which determined the annular plane, were marked manually. Then we depicted the inner contour at the level of annulus, left ventricular outflow tract (LVOT; 4 mm below the annulus), and sinotubular junction (STJ); and then the area, perimeter, maximum diameter, and minimum diameter could be calculated automatically. At the level of the ascending aorta, the maximum diameter and minimum diameter were measured manually. Mean diameter was calculated as (maximum diameter + minimum diameter)/2. The eccentricity index was calculated as (1 – minimum diameter/maximum diameter) × 100%. STJ height was measured on the central line between STJ and annulus automatically. The type of native aortic valve was identified visually in the short-axis view of the aortic root. The volume of calcification was automatically calculated for the aortic root and each coronary cusp. The inferior margins of both coronary ostia were marked manually in the long-axis view, and then the coronary heights would be generated automatically. The aortic root angle was also measured automatically once the annular plane was determined. AL ratio was defined as annulus perimeter/LVOT perimeter.

### Statistical Analysis

Continuous variables were presented as mean with standard deviation or median with interquartile range and were compared using Students *t*-test or the Mann–Whitney *U* test, respectively. Categorical variables were presented as percentages and were compared using chi-squared or Fisher's exact test. The baseline, MDCT, and procedural variables with significant difference between two groups were analyzed by Spearman's correlation and then selected in a stepwise forward conditional regression model. The variables generated by stepwise regression were introduced in the final binary multivariate logistic regression model along with anatomical variables of interest (bicuspid aortic valve and aortic root calcification). The receiver operating characteristic (ROC) curve analysis was performed to estimate the discriminative power and to identify the cutoff value of independent predictors for VM. All tests were two-tailed, and *p* < 0.05 was considered significant. All statistical analyses were performed using SPSS version 24.0 (SPSS Inc., Chicago, IL, USA).

## Results

From April 2016 to February 2020, 203 consecutive patients underwent TAVR with Venus A-Valve in five medical centers across Guangdong, China; 23 (11.3%) had VM, and all of them were valve migration toward the left ventricle. The incidence of VM was lower in patients with tricuspid aortic valve as compared with those with bicuspid aortic valve (7.1 vs. 16.7%, *p* = 0.032, Appendix 2). A total of 61 patients (30.0%) met the criteria of optimal position of the prostheses. Thus, a total of 84 patients were included in this analysis.

The baseline characteristics are shown in Table 1. The percentage of male was higher (*p* = 0.039), and the left ventricular ejection fraction was lower (*p* = 0.033) in patients with VM. The MDCT characteristics were shown in Table 2. In patients with VM, the dimensions of the annulus, LVOT, and STJ were generally larger; the left main coronary artery was higher (*p* = 0.031); and AL ratio (*p* = 0.003) was smaller. The procedural characteristics are shown in Table 3. The sizes of prostheses were significantly different between two groups (*p* = 0.009), and the prosthesis perimeter/LOVT perimeter ratio was lower (*p* = 0.026) in the malposition group. Correlation analysis showed that all the variables with significant difference between two groups were strongly correlated to either AL ratio or STJ height. Thus, only these two anatomical variables were selected in the stepwise regression, and both of them were found to be independent predictors of VM.

**Table 1:** Baseline characteristics.

	**Optimal position**	**Malposition**	***p***
	**(*n* = 61)**	**(*n* = 23)**	
**Clinical variables**			
Age, years	74.52 ± 6.98	71.26 ± 9.12	0.083
Male sex	27 (44.3)	16 (69.6)	**0.039**
Body mass index, kg/m^2^	23.12 ± 4.65	23.62 ± 2.94	0.662
NYHA class III or IV	34 (55.7)	16 (69.6)	0.250
STS score, %	3.13 (1.97–5.70)	2.53 (1.26–4.71)	0.191
Creatinine > 2 mg/dl	7 (11.5)	2 (8.7)	1.000
Hypertension	30 (49.2)	11 (47.8)	0.912
Diabetes	14 (23.0)	4 (17.4)	0.798
Coronary artery disease	26 (42.6)	9 (39.1)	0.772
Previous percutaneous coronary intervention	11 (18.0)	3 (13.0)	0.827
Previous myocardial infarction	2 (3.3)	1 (4.3)	1.000
Peripheral artery disease	12 (19.7)	2 (8.7)	0.381
Previous stroke	3 (4.9)	4 (17.4)	0.161
Chronic obstructive pulmonary disease	3 (4.9)	2 (8.7)	0.611
Atrial fibrillation	14 (23.0)	4 (17.4)	0.798
Permanent pacemaker	1 (1.6)	0 (0)	1.000
**Echocardiographic variables**			
Aortic valve area, cm^2^	0.64 ± 0.21	0.80 ± 0.15	0.052
Mean transaortic gradient, mmHg	58.09 ± 19.58	53.03 ± 23.45	0.330
Left ventricular ejection fraction, %	59.00 (44.50–66.00)	42.00 (29.00–63.00)	**0.033**
Moderate-to-severe aortic regurgitation	25 (41.0)	11 (47.8)	0.626

*Data are presented as mean ± standard deviation, median (interquartile range), or n (%)*.

*NYHA, New York Heart Association; STS, Society of Thoracic Surgeons*.

*Bold values indicates p < 0.05*.

**Table 2:** MDCT characteristics.

	**Optimal position**	**Malposition**	***p***
	**(*n* = 61)**	**(*n* = 23)**	
**Annulus**			
Area, mm^2^	437.60 (379.35–480.30)	522.80 (444.40–608.20)	**0.001**
Perimeter, mm	75.66 ± 6.51	83.02 ± 10.08	** <0.001**
Maximum diameter, mm	26.68 ± 2.56	29.23 ± 2.87	**0.001**
Minimum diameter, mm	20.87 ± 2.02	22.92 ± 2.84	** <0.001**
Mean diameter, mm	23.60 (22.25–25.00)	26.10 (24.20–28.00)	**0.001**
Eccentricity index, %	21.49 ± 6.38	21.30 ± 6.03	0.903
**LVOT**			
Area, mm^2^	428.30 (375.45–502.80)	580.30 (468.20–654.20)	** <0.001**
Perimeter, mm	77.02 ± 8.41	88.14 ± 11.60	** <0.001**
Maximum diameter, mm	27.30 (25.50–29.75)	31.00 (28.10–34.30)	**0.001**
Minimum diameter, mm	20.10 (18.90–22.00)	22.50 (20.60–25.00)	**0.002**
Mean diameter, mm	24.00 (22.35–25.75)	27.40 (24.30–29.60)	** <0.001**
Eccentricity index, %	25.00 (23.00–31.00)	27.00 (21.00–34.00)	0.488
**STJ**			
Height, mm	20.10 (18.40–22.98)	23.90 (19.80–27.10)	**0.011**
Area, mm^2^	673.30 (569.40–782.10)	765.30 (636.85–912.05)	0.064
Perimeter, mm	92.30 (84.90–99.60)	98.70 (90.15–107.95)	0.061
Maximum diameter, mm	31.12 ± 4.01	32.95 ± 5.04	0.081
Minimum diameter, mm	28.20 (25.53–29.65)	29.30 (26.40–33.00)	0.092
Mean diameter, mm	29.30 (26.65–31.30)	31.40 (28.45–33.95)	**0.048**
Eccentricity index, %	8.00 (6.00–10.00)	9.00 (5.50–12.50)	0.471
**Ascending aorta**			
Maximum diameter, mm	37.07 ± 5.34	38.30 ± 4.56	0.340
Minimum diameter, mm	35.20 (32.20–38.90)	35.20 (32.95–41.13)	0.608
Mean diameter, mm	36.10 (33.15–39.70)	35.90 (33.58–40.23)	0.812
Eccentricity index, %	3.00 (1.00–5.00)	4.00 (2.00–6.00)	0.470
**Types of aortic valve**			0.358
Tricuspid	28 (45.9)	8 (34.8)	
Bicuspid	33 (54.1)	15 (65.2)	
Types of bicuspid aortic valve			0.419
Type 0	15 (45.5)	8 (53.3)	
Type 1 RC-LC	11 (33.3)	5 (33.3)	
Type 1 RC-NC	5 (15.2)	0 (0)	
Type 1 LC-NC	2 (6.1)	2 (13.3)	
**Calcification volume**			
Aortic root calcification, mm^3^	577.25 (362.93–961.75)	675.20 (336.90–1,044.40)	0.737
NC calcification, mm^3^	230.15 (100.33–391.23)	115.30 (55.60–342.30)	0.196
RC calcification, mm^3^	213.10 (102.60–334.28)	204.20 (129.80–342.30)	0.714
LC calcification, mm^3^	132.55 (70.43–274.48)	218.80 (54.70–338.30)	0.373
**Others**			
Right coronary artery height, mm	16.77 ± 4.06	18.35 ± 3.32	0.098
Left main coronary artery height, mm	13.20 ± 3.40	15.90 ± 5.31	**0.031**
Aortic root angle, °	51.84 ± 10.68	54.48 ± 13.27	0.348
AL ratio	0.98 (0.95–1.03)	0.95 (0.92–0.97)	**0.003**

*Data are presented as mean ± standard deviation, median (interquartile range), or n (%)*.

*AL, annulus perimeter/LVOT perimeter; LC, left coronary cusp; LVOT, left ventricular outflow tract; MDCT, multidetector computed tomography; NC, non-coronary cusp; RC, right coronary cusp; STJ, sinotubular junction*.

*Bold values indicates p < 0.05*.

**Table 3:** Procedural characteristics.

	**Optimal position**	**Malposition**	***p***
	**(*n* = 61)**	**(*n* = 23)**	
Anesthesia			1.000
General	60 (98.4)	23 (100)	
Local	1 (1.6)	0 (0)	
Access route			1.000
Transfemoral	56 (91.8)	22 (95.7)	
Transcarotid	5 (8.2)	1 (4.3)	
Prostheses size			**0.009**
23 mm	18 (29.5)	3 (13.0)	
26 mm	32 (52.5)	9 (39.1)	
29 mm	11 (18.0)	8 (34.8)	
32 mm	0 (0)	3 (13.0)	
Prosthesis perimeter/Annulus perimeter	1.07 ± 0.08	1.04 ± 0.08	0.195
Prosthesis perimeter/LOVT perimeter	1.05 (1.01–1.14)	1.02 (0.90–1.07)	**0.026**

*Data are presented as mean ± standard deviation, median (interquartile range), or n (%)*.

*LVOT, left ventricular outflow tract*.

*Bold values indicates p < 0.05*.

**Figure 2:**
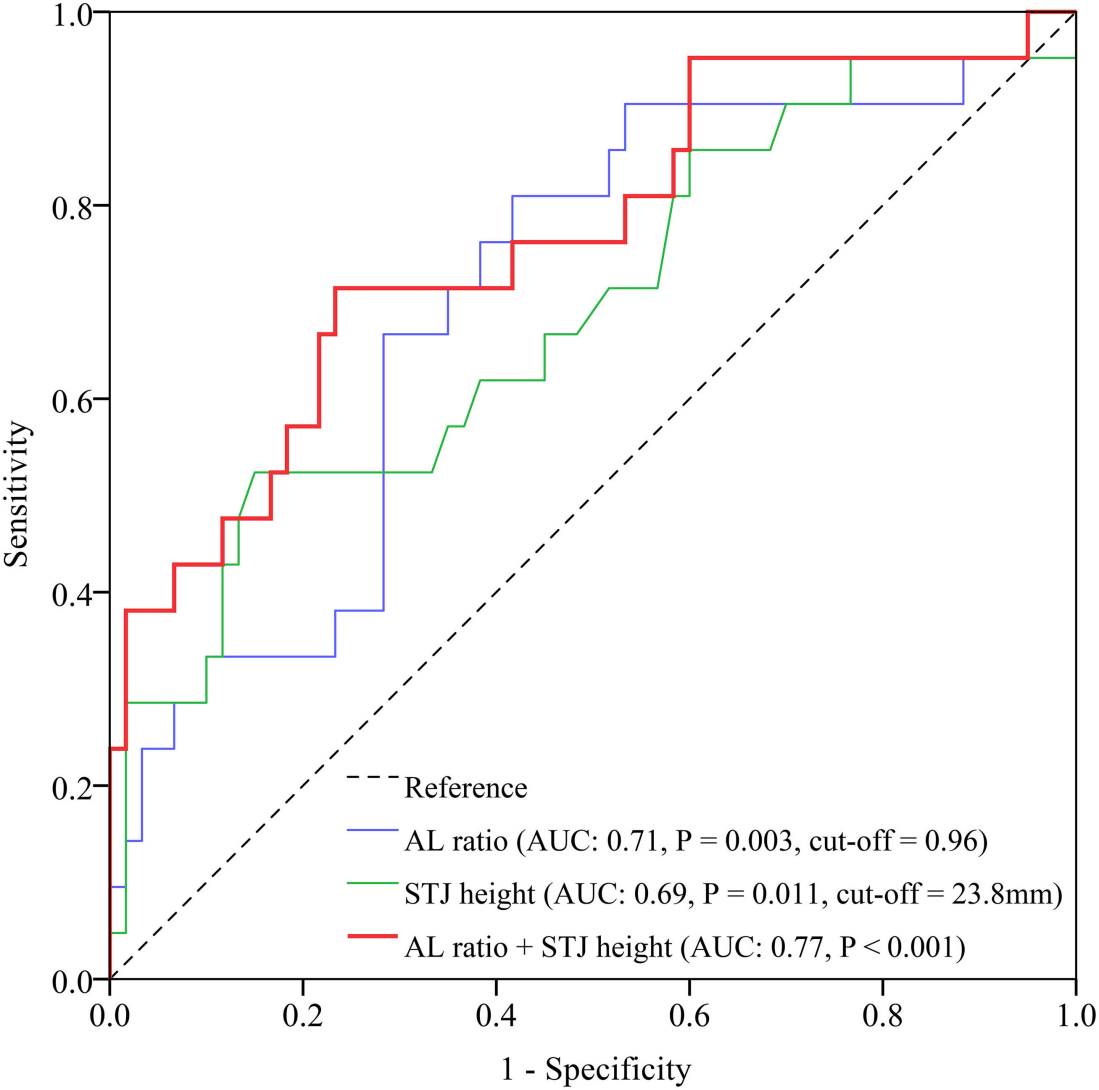
ROC curves of AL ratio, STJ height, and the combination of both for prediction of valve malposition. AL, annulus perimeter/left ventricular outflow tract perimeter; AUC, area under the curve; ROC, receiver operating characteristic; STJ, sinotubular junction.

The ROC curve indicated a moderate strength of AL ratio to predict VM [area under the curve (AUC) 0.71, 95% confidence interval (CI) 0.59–0.84, *p* = 0.003, Figure 2]. The cutoff value of AL ratio was 0.96 with a sensitivity of 70% and a specificity of 71%. The ROC curve showed a weak strength of STJ height to predict VM (AUC 0.69, 95% CI 0.55–0.83, *p* = 0.011, Figure 2). The cutoff value of STJ height was 23.8 mm with a sensitivity of 52% and a specificity of 85%. The combination of both predictors revealed a higher predictive value of VM (AUC 0.77, 95% CI 0.64–0.89, *p* < 0.001, Figure 2).

In multivariate analysis (Table 4), AL ratio <0.96 [odds ratio (OR) 3.98, 95% CI 1.31–12.14, *p* = 0.015] and STJ height ≥23.8 mm (OR 4.63, 95% CI 1.48–14.46, *p* = 0.008) were both strong independent anatomical predictors of VM. The presence of both predictors was associated with a very high risk of VM (OR 10.67, 95% CI 2.45–46.57, *p* = 0.002).

**Table 4:** Univariate and multivariate analyses for anatomical predictors of valve malposition.

**Variables**	**Univariate OR (95% CI)**	***p***	Multivariate OR (95% CI)^*^	***p***
AL ratio <0.96	4.48 (1.62–12.41)	0.004	3.98 (1.31–12.14)	0.015
STJ height ≥23.8 mm	5.30 (1.80–15.62)	0.003	4.63 (1.48–14.46)	0.008
AL ratio <0.96 and STJ height ≥23.8 mm	10.31 (2.44–43.66)	0.002	10.67 (2.45–46.57)	0.002

*AL, annulus perimeter/left ventricular outflow tract perimeter; CI, confidence interval; OR, odds ratio; STJ, sinotubular junction*.

* *Bicuspid aortic valve and aortic root calcification were included in multivariate regression model*.

There was no mortality at 30 days after TAVR in the included population. The rate of moderate-to-severe paravalvular regurgitation was higher in patients with VM (26.1 vs. 4.9%, *p* = 0.011) than in those with optimal position of the prostheses. No myocardial infarction was observed. The incidences of disabling stroke, major bleeding, major vascular complications, acute kidney injury (stage 2 or 3), permanent pacemaker implantation, and new-onset atrial fibrillation were all comparable between the two groups (Table 5).

**Table 5:** Clinical outcomes at 30 days after TAVR.

	**Optimal position**	**Malposition**	***p***
	**(*n* = 61)**	**(*n* = 23)**	
Death	0 (0)	0 (0)	NA
Disabling stroke	1 (1.6)	0 (0)	1.000
Myocardial infarction	0 (0)	0 (0)	NA
Major bleeding	5 (8.2)	3 (13.0)	0.678
Major vascular complications	3 (4.9)	0 (0)	0.558
Acute kidney injury (stage 2 or 3)	3 (4.9)	0 (0)	0.558
Permanent pacemaker implantation	7 (11.5)	0 (0)	0.182
Moderate-to-severe paravalvular regurgitation	3 (4.9)	6 (26.1)	**0.011**
New-onset atrial fibrillation	6 (9.8)	2 (8.7)	1.000

*Data are presented as n (%)*.

*NA, not available; TAVR, transcatheter aortic valve replacement*.

*Bold values indicates p < 0.05*.

## Discussion

**Figure 3:**
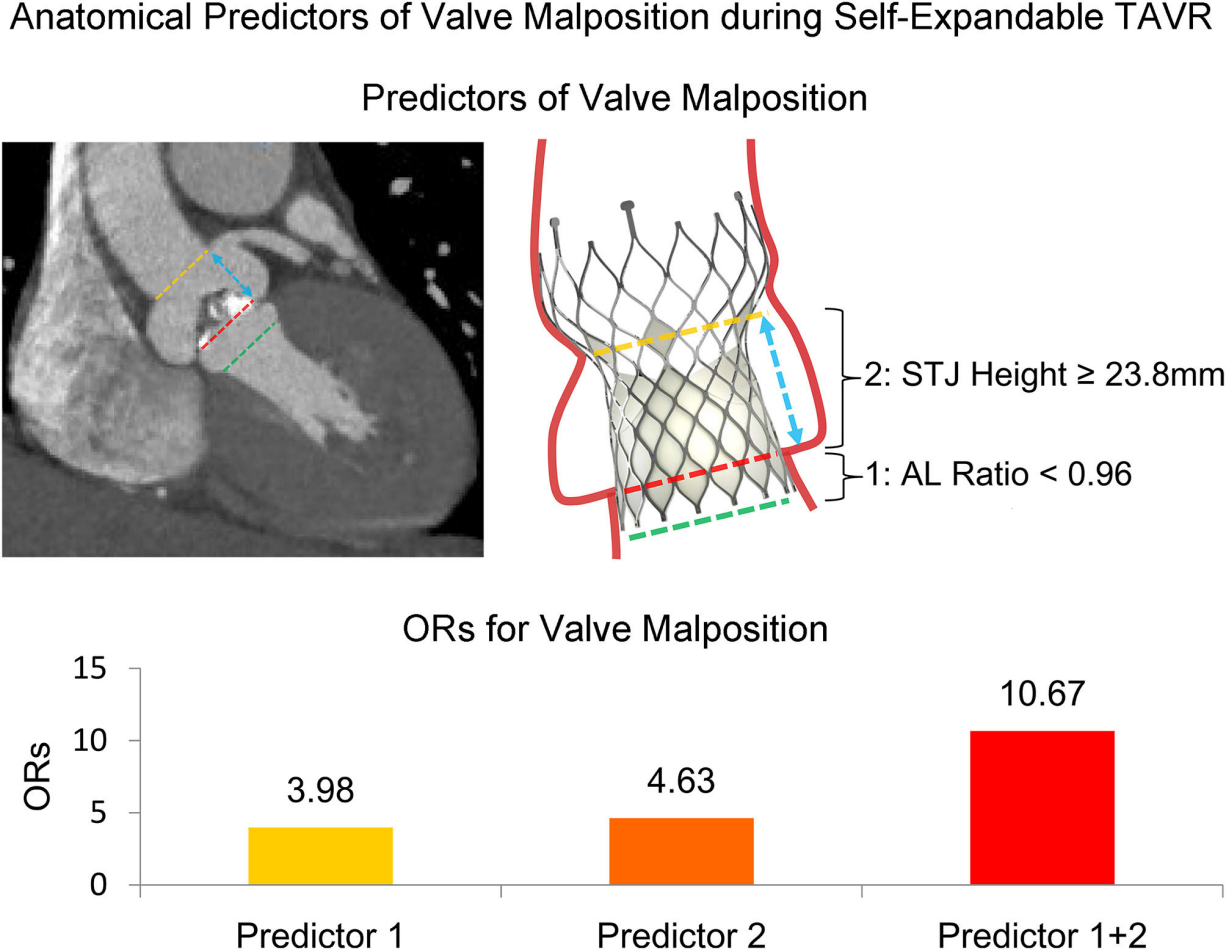
Anatomical predictors of valve malposition during self-expandable transcatheter aortic valve replacement. The dash lines indicate the planes of STJ, annulus, and LVOT. The arrow indicates STJ height. AL, annulus perimeter/LVOT perimeter; LVOT, left ventricular outflow tract; OR, odds ratio; STJ, sinotubular junction; TAVR, transcatheter aortic valve replacement.

In this multicenter retrospective study of TAVR, we utilized the modified definition of optimal position and malposition of the self-expandable Venus A-Valve; and we compared the baseline, MDCT, and procedural characteristics between those two groups. We determined that AL ratio <0.96 and STJ height ≥23.8 mm were both strong independent anatomical predictors of VM during self-expandable TAVR (Figure 3).

In clinical practice, VM could account for a majority of acute paravalvular regurgitation during TAVR and may necessitate implantation of a second prosthesis (10). However, the anatomical feature associated with VM has not been extensively investigated, particularly in a population with high calcium burden (6, 11). The recent TRAVEL registry has shed some new light on this topic and reported that the self-expandable valve was more susceptible to VM than the balloon-expandable valve in TAVR (7). Importantly, the study of new-generation devices also found a higher rate of implantation of a second valve with the self-expandable Evolut R than with the balloon-expandable Sapien 3 (2.7 vs. 0.5%) (12). The higher risk of VM during self-expandable TAVR advocated a further investigation of the anatomical predictors of VM in this setting.

To our knowledge, this is the first report that the AL ratio and STJ height are related to VM. The presence of both AL ratio <0.96 and STJ height ≥23.8 mm can increase the risk of VM at ~10-fold. Therefore, we propose a concept of the “conical LVOT,” which indicates a larger LVOT than the annulus. It is reasonable that a “conical LVOT” may not provide enough upward support force for the prosthesis during the course of release, which could prevent malposition of the prosthesis to the ventricle. It is also explainable that a higher STJ usually accompanies a larger sinus of Valsalva and therefore has a weaker radial force for the outflow of the prosthesis after release. While only the Venus A-Valve was included in this study, the AL ratio and STJ height might be helpful in evaluating the risk of VM when using other self-expandable valves with similar geometric design and releasing process (e.g., Medtronic CoreValve).

Some potentially important risk factors discovered by previous studies were not reproduced in our study, which could be the results of limited statistical power. On the one hand, bicuspid aortic valve was found to be an independent predictor (OR 3.43, *p* < 0.001) of VM in the TRAVEL registry (7). Similarly, the rate of bicuspid aortic valve was higher in the malposition group than in the optimal position group in our study, but without significant difference (65.2 vs. 54.1%, *p* = 0.358). On the other hand, in a sub-study of the PARTNER trial, heavy leaflet calcification was identified as an important cause of valve embolization or valve-in-valve procedure (6), which was not found in our study. As the problem of bicuspid anatomy and severe calcification was prominent in Chinese patients (8), both of these two variables were introduced in the final multivariate regression model to minimize the underlying bias.

The incidence of VM requiring a second prosthesis was around 11% in a Chinese population with Venus A-Valve in this study, which is much higher than that (3~4%) in western population with CoreValve (13, 14). This may be attributed to the more complex anatomy and higher degree of calcification of the aortic root in Chinese population (8) as well as a higher radial force at the inflow section of Venus A-Valve, which could enhance the pushing force downward during releasing of the prosthesis and cause VM in complicated cases (9). It has been reported that the new-generation repositionable self-expandable Evolut R and Evolut PRO have lower rates of valve migration, moderate-to-severe paravalvular regurgitation, and implantation of a second prosthesis than the earlier generation CoreValve (15–17). Similarly, it is anticipated that the second-generation repositionable Venus A-Plus Valve will likely have a better performance in terms of VM (18).

As VM relates to clinical outcome, while there was no mortality at 30 days after TAVR in our study, the rate of moderate-to-severe paravalvular regurgitation was higher in patients with VM, which was in accordance with the TRAVEL registry (7). Although the implantation of a second valve can be a safe and effective therapeutic option (13, 14), VM has been related to increased morbidity and mortality (6, 7). Therefore, we hope that the new anatomical predictors of VM could further improve the outcomes after TAVR.

### Study Limitations

There were several limitations in this study. First, there can be inherent bias as a retrospective observational study with a relatively small sample size. Second, the MDCT data were not adjudicated by a core laboratory. Finally, the ORs for predictors of VM might be overestimated to some extent because the patients with suboptimal position of the prostheses were excluded (a preliminary analysis of all patients failed to reveal any predictors, while the results of analyses in all patients using different grouping and in patients with tricuspid aortic valve were basically consistent to our finding, see Appendix 2). However, these results should be valued since the MDCT and angiography were both evaluated by two independent investigators, and multivariate regression model was applied. Future study should focus on the next-generation self-expandable valves to further corroborate our findings.

## Conclusions

In this multicenter retrospective study, we found that AL ratio <0.96 and STJ height ≥23.8 mm were strong independent anatomical predictors of VM during self-expandable TAVR in a Chinese population with high risk of VM (because of high percentage of bicuspid anatomy and severe calcification of the aortic valves). These results needed further corroboration in the next-generation self-expandable valves.

## Data Availability Statement

The raw data supporting the conclusions of this article will be made available by the authors, without undue reservation.

## Ethics Statement

The studies involving human participants were reviewed and approved by Research Ethics Committee of Guangdong Provincial People's Hospital. Written informed consent for participation was not required for this study in accordance with the national legislation and the institutional requirements.

## Author Contributions

JLi, YS, and SZ contributed equally to study design, data acquisition, statistical analysis, and drafting of the manuscript. JLu approved the submission of the final version. GL, HD, MF, YM, YL, HL, ZX, LZ, YC, RF, and DL contributed greatly to the revision of the manuscript. All authors contributed to the article and approved the submitted version.

## Conflict of Interest

The authors declare that the research was conducted in the absence of any commercial or financial relationships that could be construed as a potential conflict of interest.

## Abbreviations

AL: annulus perimeter/left ventricular outflow tract perimeter
AUC: area under the curve
CI: confidence interval
LVOT: left ventricular outflow tract
MDCT: multidetector computed tomography
OR: odds ratio
ROC: receiver operating characteristic
STJ: sinotubular junction
TAVR: transcatheter aortic valve replacement
VM: valve malposition.
