# Supplementary material for: Anatomical Predictors of Valve Malposition During Self-Expandable Transcatheter Aortic Valve Replacement
Source: Front Cardiovasc Med. 2021 Jul 12;8:600356. doi: 10.3389/fcvm.2021.600356 (PMC8311434; doi:10.3389/fcvm.2021.600356)
Supplement: Supplementary file 1 [file Data_Sheet_1.docx]

**Appendix**

**Appendix 1: Different Sizes of Venus A-Valve**


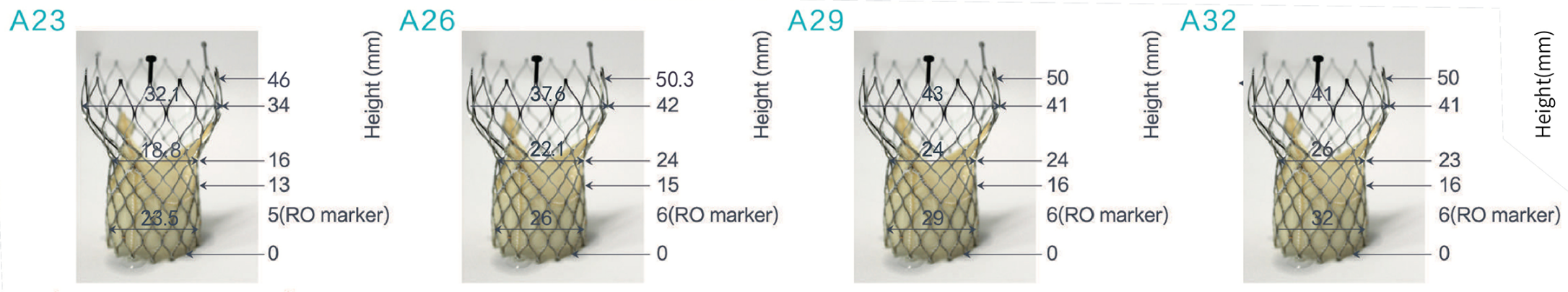


A23, A26, A29 and A32 represent Venus A-Valves with a diameter of 23, 26, 29 and 32 mm at the level of RO markers, respectively.

RO markers, which stand for radiopaque markers, are located at half a cell (5 to 6 mm) above the inflow end, indicating the optimal landing zone.

**Appendix 2 Analyses in Different Patients using Different Grouping**

**Appendix 2.1: Analysis of Anatomical Characteristics in All Patients (Optimal Position vs. Suboptimal Position and Malposition, N=203)**

|  | Optimal position | Suboptimal position and malposition | P value |
| --- | --- | --- | --- |
|  | (n = 61) | (n = 142) |  |
| **Annulus** |  |  |  |
| Perimeter, mm | 75.66 ± 6.51 | 77.68 ± 9.07 | 0.076 |
| Eccentricity index, % | 21.52 ± 6.41 | 21.88 ± 6.19 | 0.709 |
| **LVOT** |  |  |  |
| Perimeter, mm | 77.02 ± 8.41 | 80.00 ± 10.80 | 0.056 |
| **STJ** |  |  |  |
| Height, mm | 20.10 (18.40-23.08) | 21.45 (19.18-24.08) | 0.054 |
| Perimeter, mm | 92.70 (84.35-99.65) | 93.70 (85.08-101.58) | 0.824 |
| **Ascending aorta** |  |  |  |
| Mean diameter, mm | 36.20 (33.18-39.65) | 35.05 (32.50-37.95) | 0.089 |
| **Types of aortic valve** |  |  | 0.223 |
| Tricuspid | 30 (49.2) | 83 (58.5) |  |
| Bicuspid | 31 (50.8) | 59 (41.5) |  |
| **Calcification volume** |  |  |  |
| Aortic root calcification, mm^3^ | 594.10 (367.78-985.50) | 552.30 (311.88-878.78) | 0.395 |
| **Others** |  |  |  |
| Aortic root angle, ° | 51.84 ± 10.68 | 51.42 ± 10.45 | 0.797 |
| AL ratio | 0.98 (0.95-1.03) | 0.97 (0.94-1.01) | 0.149 |
| Prosthesis perimeter / Annulus perimeter | 1.07 ± 0.08 | 1.07 ± 0.10 | 0.688 |
| Prosthesis perimeter / LOVT perimeter | 1.04 (0.98-1.13) | 1.04 (0.98-1.11) | 0.901 |

Data were presented as mean ± standard deviation, median (interquartile range) or n (%). For the sake of simplicity, only essential anatomical characteristics were shown here. AL = annulus perimeter / LVOT perimeter; LVOT = left ventricular outflow tract; STJ = sinotubular junction.

**Conclusion: We failed to reveal any anatomical predictors when comparing patients with optimal position and those with non-optimal position (i.e. suboptimal position and malposition) of the prosthesis. We chose to exclude the patients with suboptimal position of the prosthesis in order to magnify the difference between the two groups (optimal position and malposition), and finally revealed the potential predictors in our manuscript.**

**Appendix 2.2: Analysis of Anatomical Characteristics in All Patients (Optimal Position and Suboptimal Position vs. Malposition, N=203)**

|  | Optimal position and suboptimal position | Malposition | P value |
| --- | --- | --- | --- |
|  | (n = 180) | (n = 23) |  |
| **Annulus** |  |  |  |
| Perimeter, mm | 76.31 ± 7.90 | 83.02 ± 10.08 | **<0.001** |
| Eccentricity index, % | 21.83 ± 6.28 | 21.31 ± 6.10 | 0.712 |
| **LVOT** |  |  |  |
| Perimeter, mm | 77.95 ± 9.45 | 88.14 ± 11.60 | **<0.001** |
| **STJ** |  |  |  |
| Height, mm | 20.80 (18.70-23.40) | 23.90 (19.80-27.10) | **0.021** |
| Perimeter, mm | 92.70 (84.70-99.90) | 98.70 (90.15-107.95) | **0.022** |
| **Ascending aorta** |  |  |  |
| Mean diameter, mm | 35.70 (32.50-38.60) | 35.80 (33.45-40.55) | 0.483 |
| **Types of aortic valve** |  |  | **0.032** |
| Tricuspid | 105 (58.3) | 8 (34.8) |  |
| Bicuspid | 75 (41.7) | 15 (65.2) |  |
| **Calcification volume** |  |  |  |
| Aortic root calcification, mm^3^ | 546.50 (323.10-868.80) | 735.70 (342.65-1052.25) | 0.425 |
| **Others** |  |  |  |
| Aortic root angle, ° | 51.17 ± 10.06 | 54.48 ± 13.27 | 0.155 |
| AL ratio | 0.98 (0.94-1.02) | 0.96 (0.92-0.97) | **0.003** |
| Prosthesis perimeter / Annulus perimeter | 1.08 ± 0.10 | 1.05 ± 0.08 | 0.193 |
| Prosthesis perimeter / LOVT perimeter | 1.05 (0.99-1.13) | 1.02 (0.96-1.04) | **0.002** |

Data were presented as mean ± standard deviation, median (interquartile range) or n (%). For the sake of simplicity, only essential anatomical characteristics were shown here. AL = annulus perimeter / LVOT perimeter; LVOT = left ventricular outflow tract; STJ = sinotubular junction.

**Conclusion: When comparing patients without malposition (including optimal position and suboptimal position) and those with malposition, there were several potential predictors. Correlation analysis showed that, except for types of aortic valve, all the other potential predictors (with P < 0.05) were strongly correlated to either AL ratio or STJ height. Finally, bicuspid aortic valve, AL ratio and STJ height were found to be independent predictors of valve malposition, when analyzing all the patients (including patients with suboptimal position).**

**Appendix 2.3: Analysis of Anatomical Characteristics in Patients with Tricuspid Aortic Valve (N=113)**

|  | Optimal position and suboptimal position | Malposition | P value |
| --- | --- | --- | --- |
|  | (n = 105) | (n = 8) |  |
| **Annulus** |  |  |  |
| Perimeter, mm | 75.94 ± 7.43 | 84.06 ± 7.31 | **0.004** |
| Eccentricity index, % | 22.20 ± 5.96 | 21.88 ± 6.63 | 0.885 |
| **LVOT** |  |  |  |
| Perimeter, mm | 77.35 ± 8.75 | 90.38 ± 9.45 | **<0.001** |
| **STJ** |  |  |  |
| Height, mm | 20.40 (18.58-23.70) | 23.90 (20.60-27.40) | **0.025** |
| Perimeter, mm | 90.60 (82.70-97.05) | 97.00 (90.90-113.40) | **0.049** |
| **Ascending aorta** |  |  |  |
| Mean diameter, mm | 34.70 (31.70-37.25) | 36.00 (32.60-41.40) | 0.330 |
| **Calcification volume** |  |  |  |
| Aortic root calcification, mm^3^ | 506.50 (302.63-763.65) | 492.85 (232.20-666.33) | 0.484 |
| **Others** |  |  |  |
| Aortic root angle, ° | 50.82 ± 10.30 | 56.13 ± 12.83 | 0.170 |
| AL ratio | 0.98 (0.95-1.02) | 0.94 (0.89-0.97) | **0.009** |
| Prosthesis perimeter / Annulus perimeter | 1.10 ± 0.09 | 1.09 ± 0.07 | 0.730 |
| Prosthesis perimeter / LOVT perimeter | 1.09 (1.03-1.14) | 1.01 (0.97-1.03) | **0.019** |

Data were presented as mean ± standard deviation or median (interquartile range). For the sake of simplicity, only essential anatomical characteristics were shown here. AL = annulus perimeter / LVOT perimeter; LVOT = left ventricular outflow tract; STJ = sinotubular junction.

**Conclusion:** **The incidence of valve malposition was lower in patients with tricuspid aortic valve as compared to those with bicuspid aortic valve (7.1% vs 16.7%, P=0.032). AL ratio and STJ height were also found to be independent predictors of valve malposition in patients with tricuspid aortic valve, consistent with what we found in the manuscript.**
